# Homelessness response: a framework for action by hospitals and healthcare systems

**DOI:** 10.3389/fpubh.2025.1642073

**Published:** 2025-11-10

**Authors:** Joy C. Liu, Catherine J. Ryan, Jeff Olivet, Emily E. Lazowy, Howard K. Koh

**Affiliations:** 1 Alameda Health System, Oakland, CA, United States; 2 Harvard T. H. Chan School of Public Health, Boston, MA, United States

**Keywords:** healthcare, homelessness, framework, public health, hospitals

## Abstract

While healthcare systems have long attempted various strategies to care for unhoused patients, the rising complexity and severity of the homelessness crisis have underscored the urgent need for systemic approaches. Such efforts are critical as current federal policies push more responsibility for homelessness prevention and response to states and localities. Few studies have identified frameworks that healthcare systems can use to guide unified responses to the homelessness. In particular, support is needed to address how healthcare systems can operate across levels beyond individual care to improve patient health. To assess current and potential best practices, we conducted a literature search on healthcare system involvement in homelessness and conducted key informant interviews with experts from healthcare systems and national and local homelessness organizations. We grouped a wide spectrum of health-system responses into ten categories: screening, Health Care for the Homeless programs, medical respite, wraparound services, medical-legal partnerships, investment in affordable housing, healthcare and housing partnerships, data sharing, anchor institutions, and implementation of federal programs. Drawing on the socioecological model, this typology provides a framework that presents the ten categories for homelessness interventions on three interconnected levels—institution-based practices, community partnerships, and public policy. It also provides a foundation for further research, financial impact analysis, and program evaluation.

## Introduction

Homelessness is an acute public health and humanitarian crisis in the United States, yet overall response from healthcare systems nationwide has been fragmented. While dedicated healthcare professionals have, over decades, offered street outreach and related services to unhoused people, the complexity and severity of the crisis now demands that all hospital and health systems adopt more systematic approaches to care, treatment, and prevention (1, 2).

Multiple factors drive this need. First, homelessness has increased; 2024 point-in-time count numbers are at their highest levels since national tracking started in 2007 (3). Those affected endure severe health inequities, as exemplified by a Massachusetts study finding all-cause mortality rates for people experiencing homelessness up to 10 times that of the general population (4). The heightened risk is driven by high burdens of chronic conditions (e.g., heart disease and cancer), infectious diseases (e.g., TB, HIV, chronic hepatitis), and mental health and substance use disorders (5–11). Second, rates of hospitalization and emergency department (ED) visits for unhoused patients greatly exceed those of the general population (12, 13). Third, healthcare systems increasingly recognize the urgency of addressing housing insecurity and other social determinants of health (SDOH) while also searching for potential cost savings (1, 14, 15).

Several recent articles document evolving healthcare system strategies to address housing insecurity and homelessness for their patients. In 2020, one of us (HK) co-authored an analysis of health-related anchor institutions, focusing on the commitment of some healthcare systems to address housing and other SDOH as part of their business strategy (16). In 2024, Garcia et al. analyzed specific hospital and health system interventions, including medical respite, screening for social needs, Housing First initiatives, and financing innovations (1). A 2025 study noted that over half of the 100 largest US health systems have some level of homelessness mitigation programs that include, for example, housing, shelter-based medical care, and coordinated services (14). Meanwhile, national non-profits working on homelessness have coordinated with healthcare systems in multiple communities (17, 18).

In 2024, the United States Interagency Council on Homelessness (USICH) released federal guidance for hospitals and healthcare systems (authored by JCL and JO). The guidance offers six best practice strategies, including delivering care outside traditional medical facilities, partnering with non-health organizations, improving data systems and data sharing on housing and homelessness, promoting supportive and affordable housing, preventing homelessness, and advancing health equity (2). Less than a year later, however, a new presidential administration has ushered in uncertainty regarding the future of federal homelessness policy (19). It is already clear that responsibility to address the homelessness crisis will fall more heavily on states and localities.

This article offers, to our knowledge, the first framework for how healthcare systems can respond comprehensively to the homelessness crisis. Based on the best publicly available data, it offers a vision of how health care organizations can employ a broad array of strategies and interventions to identify levels of engagement and unify diverse approaches. The framework can also help organize future research efforts for evaluation.

## Methods

To identify the most promising interventions, we first identified relevant articles in the peer-reviewed literature. Using individual search terms and combinations of terms (e.g., “housing instability,” “homelessness,” “healthcare systems,” “hospitals,” “housing first,” “affordable housing”), two reviewers (JL and CR) searched databases including PubMed, Google Scholar, Policy File, Policy Commons, and Think Tank Search for published articles (January 2000–March 2025) relating to any healthcare system’s activity addressing homelessness. We then conducted extensive internet searches of the substantial “grey literature” outside of academic databases, employing key items like “homelessness” and “health care systems” and “housing first” and “affordable housing.” that described American healthcare organizations engaged in activities related to homelessness. Finally, we also conducted 12 key informant interviews with recognized healthcare leaders and local and government partners across the country on perceived reasons for involvement, best practices, and potential areas for increased involvement in addressing homelessness. The 30-min interviews deployed a semi-structured interview guide using snowball sampling.

**Figure 1:**
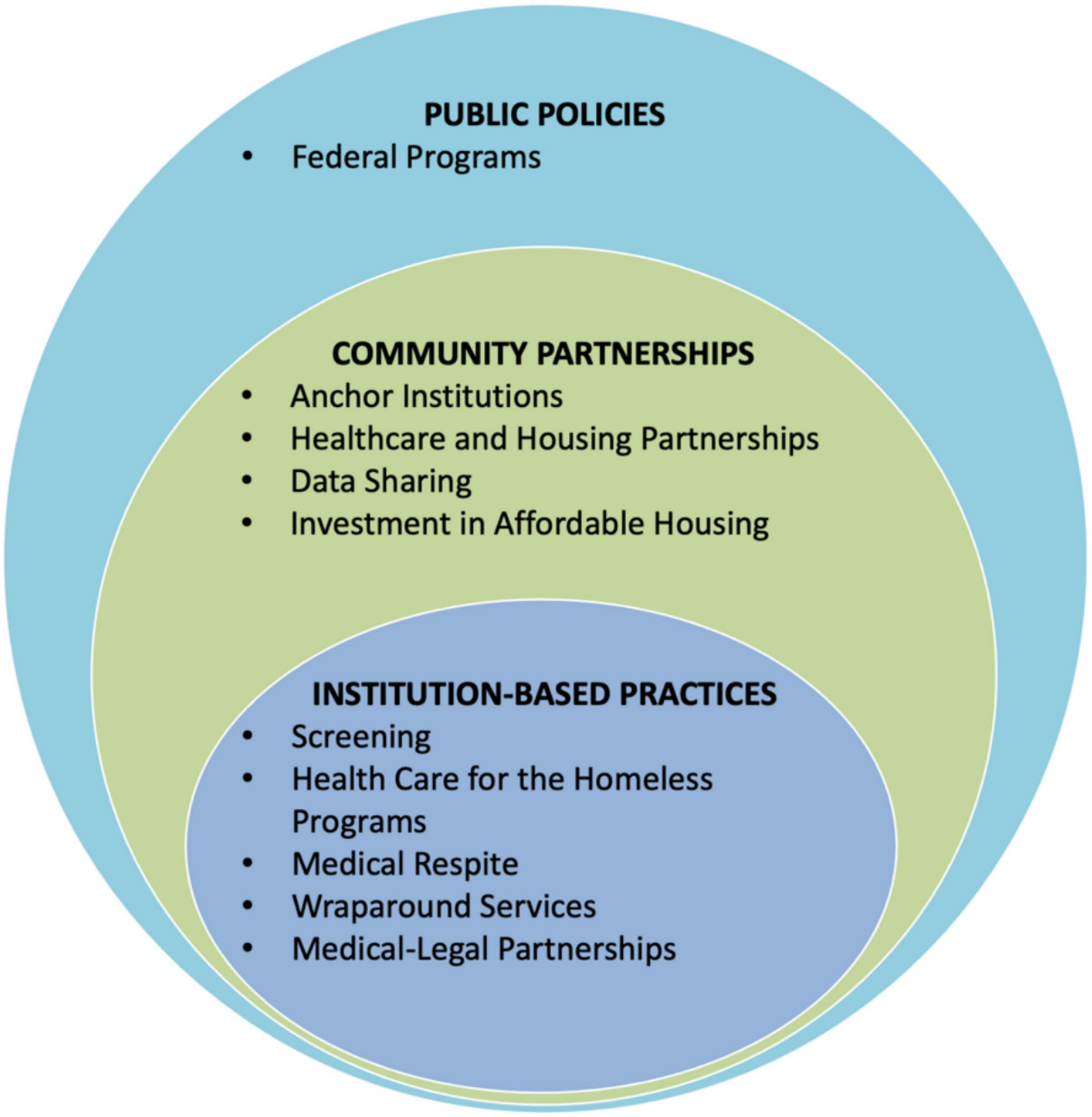
Framework for a comprehensive healthcare response to homelessness.

Synthesizing over 180 items from the white and grey literature as well as information from key informant interviews, we grouped each item into at least one of ten categories (Table 1). The categories represent the building blocks of a broader three-level framework of health system involvement (Figure 1).

**Table 1:** Descriptions and examples of healthcare system involvement in addressing homelessness.

Category	Description	Activities and examples
Institution-based practices		
Screening	Systematic screening and record keeping of patients who are housing insecure or at risk of homelessness	Activities include: Screening patients in a clinical setting for housing needs Predictive modeling using screening data to identify patients at risk for homelessness
Health Care for the Homeless (HCH)	Community-based initiatives (and federally qualified health centers) using collaborative, interdisciplinary team approach	Examples include: National Health Care for the Homeless Council provides unified resources for HCH programs 300 + programs across the U. S. serving nearly 1 million patients annually including some well-known and studied examples: Boston Healthcare for the Homeless Program (BHCHP) Streamlined Unified Meaningfully Managed Interdisciplinary Team (SUMMIT), Portland, Oregon
Medical respite	Short-term shelter and medical care for people experiencing homelessness who have recently been discharged from traditional medical care	Activities include: 24-h access to beds Meals Wellness checks Care coordination and transportation Housing intervention services
Wraparound services	Assessing, planning, and facilitating access to health and social services, housing, mental health, substance use care	Activities include: Counseling Multi-disciplinary social care teams Referral to treatment or prevention programs Follow-up programs (e.g., staff check-ins) Patient navigators Coordination with housing advocates & other community supports Critical Time Intervention
Medical-Legal Partnerships	Integrating legal expertise into healthcare settings to provide patients with legal services related to housing	Kaiser Permanente Medical-Legal Partnership Legal outreach at VA medical centers National Center for Medical-Legal Partnerships
Community partnerships		
Healthcare and housing partnerships	Direct partnership or coalition building between healthcare systems and housing organizations	Examples include: California Health Care and Homelessness Learning Collaborative, launched by the California Health Care Foundation and the Center for Health Care Strategies Detroit Healthy Housing Center, a partnership between healthcare & housing organizations providing medical respite and other types of wraparound care
Anchor institutions	Healthcare systems invest in their communities as a “way of doing business”	Activities include: Healthcare system investment in affordable housing, community land trusts Examples include: Mayors and CEOS for US Housing Investment, a bipartisan coalition advocating for housing policy Chicago’s Healthcare Anchor Network and West Side United are collaboratives including hospitals that coordinate investments in affordable housing Health Anchor Network and the Democracy Collaborative resources
Data sharing	Record sharing/coordination with social service agencies (11)	Activities include: Agreements coordinating electronic health records and information management systems Cross-access to patient data by hospitals and social service agencies Data sharing toolkits
Investment in affordable housing	Investment by healthcare systems in affordable housing or related community services in communities served	Examples include: CVS Health: $1.3 Billion UnitedHealth Group: Over $1 Billion Kaiser Permanente: $400 Million Combined stated goal number of resulting housing units: 26,000 units
Public policies		
Federal programs	Implemented, collaborated, or interacted with federal programs and policies	Examples include: VA-Supportive Services for Veterans and Families (SSVF) programs provide housing assistance, case management, and supportive services to help very low-income Veteran families stay in or obtain stable housing. VA-HUD Supportive Housing (HUD VASH) program provides permanent supportive housing to veterans experiencing homelessness Medicaid 1,115 Waivers targeting SDOH and allowing Medicaid to cover housing costs and related social services

## Results

Based on results from the literature reviews and key informant interviews, we developed a framework through which hospitals and healthcare systems can create a more unified and comprehensive response to homelessness. Building on the socioecological model, the framework presented in Figure 1 encourages coordinated change at three levels—institution-based practices, community partnerships, and public policy (20). By highlighting the inherent links between these layers, this framework illustrates how healthcare systems can operate across levels beyond individual care to improve patient health. It gives health care systems a blueprint for building a comprehensive response, starting with activities closest to the central activity of patient care and then expanding outward to organizational collaboration (institutions and communities) and policy change.

### Institution-based practices

We identified various forms of team-based practices that hospitals and other healthcare systems have used to address homelessness. With the addition of data linking each intervention to specific health or housing outcomes, these represent initial steps healthcare systems can use to build a comprehensive response to homelessness.

#### Screening

Some healthcare systems have used screening protocols to identify patients who are housing insecure or homeless and provide them with necessary support and services. At present, the US Department of Veterans Affairs (VA) has had the most experience with developing, and testing a standardized instrument—the Homelessness Screening Clinical Tool—that identifies veteran families experiencing housing instability and at risk of homelessness (21, 22). Montgomery et al. found that when the VA’s national Homelessness Screening Clinical Reminder launched, between October 1, 2012 and January 10, 2013, 1,422,038 veterans were presented with the screener, with 2.1% of viable respondents screening “positively” for a risk of homelessness (21). Since then, Byrne et al. tested predictive modeling using medical record data to further hone the VA screener process (22). At the community level, Houston’s Ben Taub Hospital has screened for, and flagged, heavy emergency department utilizers to help address social, including housing, need, connecting identified patients with community organizations and community health workers or social workers prior to discharge (23).

Legislation in California in 2019 prompted hospitals to screen patients for homelessness, keep records of discharge planning, and offer food, clothing, and appropriate referrals (24). After this legislative change, qualitative studies found that staff reported increased consistency in documentation and service delivery—although they also expressed concerns regarding accuracy and insufficient availability of resources to respond to patient needs (25–27). Patients have generally responded positively to being asked about housing in the ED, even if many had not initially considered the ED a place to receive help with housing (28).

#### Health Care for the Homeless (HCH)

Established in 1985, the HCH program involves a collaborative, interdisciplinary team-based approach for patients experiencing homelessness. The National Healthcare for the Homeless Council (NHCHC), founded in 1986, coordinates, convenes, and supports HCH projects across the country, which today number over 300 sites serving 940,000 patients each year (29).

In one example, the Boston Health Care for the Homeless Program (BHCHP) provides essential services for 11,000 + individuals in settings including street medicine, primary care, subspecialty care, and post-acute care. A recent study found that adding patient navigators to standard-of-care lung cancer screening for BHCHP patients increased low-dose CT scans 4.7-fold (30). Similarly, a randomized controlled study involving SUMMIT, the Streamlined Unified Meaningfully Managed Interdisciplinary Team, a federally qualified health center team in Portland, Oregon, found that interdisciplinary team care increased primary care use and improved patient wellbeing, although it did not meaningfully change hospital or ED use (31, 32). Shared decision-making, continuity of care, integration of social services, and services that reach beyond traditional primary care clinics positively impact patient experience (33, 34). Many HCHs remain free-standing programs that could have broader impact through closer collaboration with hospitals and health systems (35).

#### Medical respite

Some healthcare systems offer short-term housing and medical care for unhoused patients too ill to be on the streets or in traditional shelters but not ill enough to require hospitalization. It bridges the gap between hospital care and shelters or provide recovery space for unsheltered people such programs could reduce future hospital admissions, readmissions, and inpatient days (36–39). Facility type varies from transitional housing to standalone facilities; common features include 24-h access to beds, three daily meals, wellness checks, care coordination, and transportation to medical appointments (40). BHCHP opened the first medical respite facility in 1985 which currently includes over 100 beds (41). Today, there are over 150 medical respite sites in the U.S (1).

Multiple studies demonstrate cost savings from medical respite programs, with average daily expenses an estimated $2,282 less per day than inpatient hospital care (42, 43). A randomized study at two large Chicago hospitals involving 470 adults with chronic diseases, two respite sites, and 10 housing agencies, found patients connected to medical respite (through hospital case managers) used the ED 24% less than other patients experiencing homelessness (44). Doran et al. also associated medical respite with improved housing outcomes, health status, and access to primary care (45).

#### Wraparound services

These services typically facilitate access to health and social services commonly centered on housing, mental health, and substance use (46, 47). Interventions are varied and include different models of case management [standard, intensive, clinical, Assertive Community Treatment (ACT), and Critical Time Intervention (CTI)], housing navigation, and care coordination. Preliminary evidence suggests that such wraparound services can decrease hospital days and ED visits, benefit housing stability, ameliorate drug and alcohol use disorders, and reduce days of homelessness (44, 48, 49).

Critical Time Intervention (CTI) is a time-limited case management model that provides support during challenging life transitions, including from a hospital into the community. A systematic review of CTI found positive impacts on housing and service engagement use outcomes (50). One randomized trial of family CTI for mothers experiencing homelessness found those who received CTI were more likely to be rapidly rehoused compared to those receiving usual services (51).

#### Medical-legal partnerships (MLPs)

MLPs integrate legal expertise into medical settings by embedding lawyers into healthcare teams to provide patients with legal services related to housing, landlord-tenant disputes, evictions, and discrimination (52). Funding generally comes from healthcare organizations, philanthropy, and legal volunteers (53). A VA study showed that participants’ legal goals were achieved in 712 out of 1,384 issues raised one such MLP (54). Veterans whose legal needs were addressed had significantly fewer symptoms of hostility, paranoia, and generalized anxiety disorder, and social outcomes—including housing status (54). However, one study noted that of 48 homeless service sites across 48 states, only 10% had MLPs despite the fact that 90% reported that their patients had civil legal needs (55).

### Community partnerships

Beyond providing direct care, individual hospitals and health systems have addressed homelessness by forming partnerships with other organizations in their communities. There remains a pressing need for additional data on their effects on patient care and best partnership practices.

#### Healthcare and housing partnerships

Several promising examples of direct partnership and building coalitions can align healthcare systems and the Continuum of Care — locally or regionally organized groups that coordinate housing and services for people experiencing homelessness. The California Health Care and Homelessness Learning Collaborative (launched in 2022) promotes cross-sector collaboration between the California Health Care Foundation and the Center for Health Care Strategies (56). In Detroit, Michigan, an integrated health and social services agency partnered with nonprofit, healthcare partners, and housing organizations (2023) to form the “Detroit Healthy Housing Center,” offering medical respite beds to 165 patients experiencing homelessness annually (57). In 2020, non-profit organizations Community Solutions and the Institute for Healthcare Improvement launched a health care and homelessness pilot that unified major health systems in five US communities: Kaiser Permanente (KP), Providence Health, Common Spirit, Sutter Health, and U.C. Davis Health (58). These organizations engage in cross-sector case conferencing, coordinating post-discharge care, and sharing data between healthcare and housing systems (59).

#### Data sharing

Hospitals and healthcare systems can unify patient care for unhoused patients by sharing data with social service agencies. In Washington County, Oregon, and Sacramento County California, numerous organizations, including KP have entered data sharing agreements across the medical system and social services continuum of care. The aim is to ensure that EHRs and the Homeless Information Management System (HMIS) collect similar data and that hospital navigators have access to HMIS systems (60). Community Solutions has developed a data sharing toolkit based on these pilot projects (60).

#### Anchor institutions

Some hospitals and health systems have committed to addressing SDOH, including housing insecurity, as part of their business mission. Such “anchor institutions” are major, place-based health institutions that commit significant financial, human, and intellectual resources to addressing social challenges. Anchor medical institutions may, for example, invest in affordable housing and community land trusts (16). Nonprofit hospitals can achieve federal, and often state, tax-exempt status by assessing community health needs and ensuring community engagement in strategies targeting social needs like housing.

As part of their anchor strategy, KP joined a bipartisan advocacy coalition—Mayors and CEOS for US Housing Investment—to advocate for housing policy (16, 61). Rush University in Chicago similarly joined the think tank Democracy Collaborative and the Civic Consulting Alliance in 2017 to develop the Healthcare Anchor Network, a national collaboration of over 75 healthcare systems investing in affordable housing in local communities (62). In 2018, Rush joined with five other hospitals to develop the West Side United collaborative focused on racial equity and coordinating affordable housing investments (62).

#### Investment in affordable housing

Healthcare systems directly invest into local housing markets. A 2020 study estimated that healthcare systems spent $1.6 billion (from 2017 to 2019) on housing-focused interventions, including affordable housing (15). The largest investors appear to be CVS Health (over $1.3 billion), UnitedHealth Group (over $800 million), and KP ($400 million) with a stated overall goal of over 26,000 new housing units across the country, including 7,000 new housing units over the last 5 years (63–65). These investments should be evaluated further by researchers and public health practitioners through analysis of publicly available impact data.

### Public policies

Healthcare systems have implemented, collaborated on, or interacted with federal programs and policies to increase affordable housing and related needs. For example, in 1992, the VA partnered with the US Department of Housing and Urban Development (HUD) to establish the HUD-VA Supportive Housing (HUD-VASH) program, providing veterans rental subsidy vouchers, case management, and other wraparound services. A 2003 randomized control study found that HUD-VASH veterans, compared to those receiving case management only or standard care, had 16 and 25% more days housed, respectively (66). HUD-VASH expanded dramatically after 2008, and has been linked to reducing veteran homelessness by 55% (67, 68). The VA’s related Supportive Services for Veteran Families (SSVF) program has been used to rapidly rehouse not only veterans but also their families, often through partnerships with community-based non-profit organizations (69).

Through Medicaid, states have had the opportunity to submit 1,115 waivers, giving them flexibility for states to reimburse for costs related to housing support and other health-related social services (not exceeding 3% of total annual Medicaid spending) (70). Most waivers were narrow in scope until the 1990s, but have since expanded and now nearly one-third target homelessness (14, 71, 72). One such pilot program in North Carolina reduced overall Medicaid spending as a result of the waiver (73).

### Other comprehensive responses

The nation has seen some examples of comprehensive responses to homelessness through healthcare systems. The most prominent ones relate to the VA through HUD-VASH and SSVF, as noted above. Outside the VA, the Harris Health System, a Texas safety-net provider serving approximately 4.7 million patients offers wraparound services for unhoused patients who frequently use EDs. Harris also partners with the Coalition for the Homeless of Houston/Harris County and community service agencies to coordinate care and provide emergency housing assistance (74). KP, an integrated healthcare system serving over 12 million patients in eight states (and the District of Columbia), has over the last decade invested in medical respite and medical-legal partnerships. Efforts include a 2021 launch of medical-legal partnerships with five medical centers across four states, with 330 closed cases during 2022–2023, 91 of them related to housing and/or utilities (75). And as noted above, KP has also joined coalition partnerships focused on housing and invested over $400 million in affordable housing (16, 61, 65).

## Conclusion

This article offers a vision for a more strategic and comprehensive approach to caring for people experiencing homelessness. In particular, it offers healthcare systems ways to operate across levels beyond individual care to improve patient health. Our multi-level intervention framework rooted in the socioecological model synthesizes existing practices into a blueprint versatile enough for application across locations, policy landscapes, and healthcare systems. The framework organizes an array of concrete opportunities for hospitals and healthcare systems to expand their role as population-health change agents at a time when federal and many state governments are stepping back from commitments to addressing housing and other SDOH.

The framework also builds upon and formalizes the previous federal guidance from USICH, offering a roadmap for hospitals and health systems to operationalize it. While we offer here a consolidation of diverse actions into one framework, relatively few services have been rigorously evaluated. As more healthcare systems engage in this work, they should generate rigorous, publicly available research on health and housing outcomes. Specific pressing and high-yield questions include: What is the return on investment for monetary commitment in affordable housing? Are there significant differences in housing outcomes after establishing healthcare and housing partnerships? What is the best way to measure and monitor outcomes? How can individual care interventions be designed to improve specific health outcomes? Since this article was informed by an initial review of current literature, we encourage a more detailed systematic review on the literature and its quality.

Healthcare researchers, policymakers, and leaders are well-positioned to begin answering these questions, as well as to create guidelines, toolkits, and other resources for continued development and learning. Further commitment will be critical to ensure a future society where no patient experiences the tragedy of homelessness.

## Data Availability

The original contributions presented in the study are included in the article/supplementary material, further inquiries can be directed to the corresponding author/s.
